# Editorial: Biomedical Data in Human–Machine Interaction

**DOI:** 10.3390/s23187983

**Published:** 2023-09-20

**Authors:** Aleksandra Kawala-Sterniuk, Grzegorz Marcin Wójcik, Waldemar Bauer

**Affiliations:** 1 Faculty of Electrical Engineering, Automatic Control and Informatics, Opole University of Technology, 45-758 Opole, Poland; grzegorz.wojcik@live.umcs.edu.pl; 2 Department of Neuroinformatics and Biomedical Engineering, Maria Curie-Sklodowska University, 20-400 Lublin, Poland; 3 Department of Automatic Control and Robotics, AGH University of Science and Technology, Al. Mickiewicza 30, 30-059 Kraków, Poland; bauer@agh.edu.pl

Analysis of biomedical data can provide useful information regarding human condition and as a result—analysis of these signals has become one of the most popular diagnostic methods.

As mentioned above, analysis of biosignals plays an important role in health monitoring, which has led into the need for design and development of new efficient methods for the processing of these signals.

Our Special Issue aimed to introduce the most recent advances in the interpretation of various biomedical data and their potential implementation in human–computer interaction. It also covered the newest methods for interpretation of various types of biomedical data, including model-based biosignal analysis, data interpretation and integration, as well as medical decision making extending the existing data processing methods and technologies for their effective application in clinical environments.

Also, as mentioned above, as the analysis of biomedical data covers a wide range of interesting topics, this Special Issue covered, in our subjective opinion, many of them.

The submitted papers covered, among others, the following areas: analysis of various types of biomedical data, e.g., electroencephalography (EEG), electromyography (EMG), electrocardiography (ECG), magnetic resonance imaging (MRI). it also covered areas strongly related with human–machine interaction, such as smart cities and smart homes; brain–computer Interfaces, human–machine Interaction, graphomotorics, and neurodegenerative disorders—diagnostics and movement disorders.

This Special Issue consists of a total of 20 papers, 4 review and 16 research works, covering a wide area of biomedical data in human–machine interaction.

The first paper [1], authored by Paerai et al. and titled “Functional living skills: a non-immersive virtual reality training for individuals with major neurocognitive disorders”, concerns the loss of functional living skills (FLS) as a result of major neurocognitive disorders (M-NCD). The paper describes and verifies the effectiveness of using virtual-reality-based training (VRT) for improving FLS in people with M-NCD. The satisfaction of the study participants was moderate to high, so the obtianed results can be considered as positive.

The next three papers [2,3,4] written by Martinek et al. are of review character and are part of a larger whole. The first one [2] titled “Advanced Bioelectrical Signal Processing Methods: Past, Present and Future Approach—Part I: Cardiac Signals” covers the most current signal processing methods of various cardiac signals, such as, inter alia, electrocardiography, vectorcardiography and fetal electrocardiography, while the second one [3] titled “Advanced Bioelectrical Signal Processing Methods: Past, Present and Future Approach—Part II: Brain Signals” describes the most current methods for analysis and processing of various brain signals, such as, among others, electroencephalography, electrocorticography or functional near-infrared spectroscopy. The last one [4], itled “Advanced Bioelectrical Signal Processing Methods: Past, Present and Future Approach—Part III: Other Biosignals”, describes the newest and most efficient signal processing methods applied for analysis of other biomedical signals, which were not described in [2], such as, inter alia, electromyography, electroneurography, electrogastrography, electrooculography, electroretinography and electrohysterography.

Another interesting paper written by Dyląg et al. [5] titled “Pilot Study on Analysis of Electroencephalography Signals from Children with FASD with the Implementation of Naive Bayesian Classifiers” presents the use of Naive Bayesian Classifiers as a non-invasive method for Fetal Alcohol Spectrum Disorder detection based on EEG signals analysis. The obtained results offered an efficiency of over 75% and were promising.

The next paper, written by Khoma et al. [6] titled “Advanced computing methods for impedance plethysmography data processing”, describes innovative solutions applied for the improvement of impedance plethysmography data in order to ensure the parameter stability and additionaly to extend the functionality of the rheographic systems. The study result was the development of three methods for respiratory artifact elimination.

Russell et al. published a paper titled “Towards Dynamic Multi-Modal Intent Sensing Using Probabilistic Sensor Networks” [7] where they focused on intent sensing, which is the ability to sense what a user wants to happen. The authors described how assistive medical devices, such as, e.g., prosthetic limbs, could benefit from intent-based control systems, which allow their faster and more intuitive control. They also claimed that issues related with the intent sensing accuracy could be improved by using multiple sensors, which enable the sensing of multiple environments. The paper concentrates on the development and testing of a sensing system under changing conditions with the use of Bayesian sensor fusion.

The next paper written by Kręcisz et al. [8] titled “Using Nonlinear Vibroartrographic Parameters for Age-Related Change Assessment in Knee Arthrokinematics” concentrates on changes in articular surfaces associated with aging, which lead to quantitative and qualitative joint motion impairment. This paper evaluates the age-related knee joint quality arthrokinematic motion using nonlinear parameters of the vibroarthrographic (VAG) signals, such as recurrence rate (RR) and multi-scale entropy (MSE). The obtained results provide additional information regarding the the nature of changes in the vibration dynamics occurring during the aging process.

Antonowicz et al. in their work [9] titled “Digital Stereotypes in HMI—The Influence of Feature Quantity Distribution in Deep Learning Models Training” proposed a concept of Digital Stereotypes which can be observed during research on quantitative overrepresentation of one class over others, and its impact on the results of the training of Deep Learning models. The paper covers a wide area of Deep Learning models, which, when properly taught with overrepresentation, may produce incorrect inferring results, similar to the stereotypes. The authors also evaluated a large dataset in various scenarios. Their results may be applied to any multiclassification applications. The paper focuses on the following areas: machine learning, feature measurement, cognitive technologies, artificial intelligence, Industry 5.0 and digital stereotypes.

In paper [10] titled “Stressor Length and the Habituation Effect—An EEG Study” and authored by Rejer et al., the authors aimed to determine whether there is a difference in response to long and short stimuli and whether the stress stimuli repeated over time can evoke a habituation effect. The authors described a cognitive experiment with eight participants, where two trays of stress-inducing stimuli of different length are presented. The very interesting result of this work is fact that no evidence to confirm (or reject) the hypothesis that stress stimuli repeated over time evoke the habituation effect was found.

Al-Bakri et al. in [11] titled “Implementation of a Morphological Filter for Removing Spikes from the Brain Signals to Improve Identification Ripples” focused on using a morphological filter in the analysis of invasively recorded brain signals—intracranial EEG (iEEG)—from patients affected with epilepsy in order to improve the identification of epilepsy-related ripples. Their method’s average sensitivity and false detection rate is significant, as it is 94% and 14%, respectively.

In paper “Age-Related Differences in Intermuscular Coherence EMG-EMG of Ankle Joint Antagonist Muscle Activity during Maximal Leaning” [12] authored by Konieczny et al., the authors focused on intermuscular synchronization, which is the one of the fundamental aspects for maintaining a stable posture. The authors tried to assess the muscle synchronization and postural stabilizer asymmetry during quiet standing and the limits of stability with the implementation of Wavelet analysis; they also evaluated intermuscular synchrony and antagonistic sEMG-sEMG (surface electromyography) coherence asymmetry in tibialis anterior and soleus muscles. The obtained results show differences in the beta and delta band oscillations between younger and older study participants in a postural task involving standing quietly and leaning forward.

Han et al. in their work “Brain Age Prediction: A Comparison Between Machine Learning Models Using Brain Morphometric Data” [13] presented machine-learning-based approaches for the purpose of abnormal aging process detection. The authors evaluated 27 machine learning models, which they applied to three independent datasets: the Human Connectome Project, the Cambridge Centre for Ageing and Neuroscience and the Information eXtraction from Images. The authors found a substantial difference in performance between models trained on the same data type; additionaly, in three datasets, the regularized linear regression algorithms achieved similar performance to that of nonlinear and ensemble algorithms. The obtained results suggest that for brain age prediction, the regularized linear algorithms are as effective as the nonlinear and ensemble algorithms. The regularized linear algorithms also enable significant computational cost reduction.

The next paper written by Łysiak et al. [14] titled “Repeatability of the Vibroarthrogram in the Temporomandibular Joints” describes the examination of sound repeatability in temporomandibular joints (TMJ) in order to investigate the repeatability of the specific vibroarthrogram (VAG) features of the the TMJ using accelerometers. The obtained results show the potential of of the vibroarthrogram features in the context of the TMJ, which would further improve the diagnosing process.

Borysiuk et al. in [15] (“Correlations between the EMG Structure of Movement Patterns and Activity of Postural Muscles in Able-Bodied and Wheelchair Fencers”) analyzed EMG data from Paralympic wheelchair fencers. The authors carried out a heuristic analysis which indicated the postural muscle significance in the movement patterns of wheelchair and able-bodied fencers, and their study proved that these muscles play a crucial role in the anticipatory postural adjustment of the trunk during technical fencing actions.

In a paper titled “Advanced Modeling and Signal Processing Methods in Brain–Computer Interfaces Based on a Vector of Cyclic Rhythmically Connected Random Processes” [16] authored by Lupenko et al., a new mathematical model of a vector of electroencephalographic signals is presented. The obtained results show that the adequacy and ability of the proposed mathematical model and methods for vector EEG processing based on the vector of cyclic rhythmically relates random processes to reflect the mental controlling actions of the BCI operator in its characteristics and the possibility of conducting a high-precision procedure for their detection are efficient. Also, the use of higher-order moment functions and their spectral images in the frequency domain as informative characteristics in Brain–Computer Interfaces systems is justified.

Anna Czmil in her paper “Comparative Study of Fuzzy Rule-based Classifiers for Medical Applications” [17] used machine-learning-based methods in medical decision support systems, which can significantly improve diagnostic accuracy and objectivity for clinical experts. In this paper, the author carried out a thorough comparison of 16 different fuzzy-rule-based algorithms, which were applied on data obtained from 12 medical datasets and real-world data. The obtained results show that the best-performing algorithm is a classifier based on fuzzy logic and gene expression programming (GPR). The author, based on her research, suggests that GPR is capable of generating concise and interpretable rules while maintaining good classification performance, and it may be a valuable algorithm for generating rules for medical data.

Khoma et al. in their paper [18] “Development of Supervised Speaker Diarization System Based On the PyAnnote Audio Processing Library” focused on audiodata diarization. The authors proposed two architectures of speaker identification systems based on a combination of diarization and identification methods operating on the segment-level basis or on group-level classification. For this study purpose, an open-source PyAnnote framework was applied. The presented research method consists of four experiments in order to select the best-performing one.

Zemla et al. in [19] titled “Investigating Impact of Guided Imagery on Stress, Brain Function, and Attention: A Randomized Trial” focused on the examination of the potential effects of guided imagery and investigation of the correlation between guided imagery, stress reduction, alpha brainwave patterns, and attentional control using standard cognitive performance assessments. The evaluation of the executive function was executed through attentional control tests, encompassing tasks such as anti-saccade, Stroop, and Go/No-go tests. Study participants engaged in a guided imagery session while their brainwave activity was recorded, followed by attentional control evaluations. The outcomes of the study offer fresh insights into the impact of guided imagery on brainwave activity, particularly in relation to attentional control. The results propose that guided imagery holds the capacity to enhance attentional control by amplifying alpha power and decreasing stress levels. Given the scarcity of existing research on the precise influence of guided imagery on attentional control, the findings of this study carry significant importance.

The last paper, authored by Pirri et al. ([20]) titled “Hearing and Seeing Nerve/Tendon Snapping: A Systematic Review on Dynamic Ultrasound Examination” is of a review character and focuses on dynamic ultradound examination. This is because nerve and tendon snapping can occur when they suddenly shift during the movement of an adjacent joint, leading to a clinically painful condition. Identifying the precise anatomical structure responsible for the snapping in various body regions can be quite challenging. In this regard, ultrasound examination, known for its advantages, particularly its ability to provide dynamic imaging, shows significant promise. However, there is currently a lack of comprehensive reviews that discuss the utilization of dynamic ultrasound examination in diagnosing nerve and tendon snapping. As a response, this article aims to present a thorough exploration of how ultrasound examination could enhance the understanding of these pathologies by visualizing and capturing the various maneuvers and movements associated with them.

As previously highlighted, delving into the analysis of biomedical data presents a formidable challenge. Yet, it is precisely this complexity that renders the endeavor captivating and intellectually stimulating. The intricate interplay of data, the need for innovative methodologies, and the potential to uncover profound insights about the human body and mind make this domain incredibly fascinating.

We strongly believe that our research topic holds the potential to captivate a wide array of readers and researchers across diverse fields, including medicine, biomedical engineering, computer engineering, control, biocybernetics and neuroscience. The intricate nature of biomedical data analysis resonates with those seeking to decipher the mysteries of human health and cognitive function. Biomedical engineers would find this topic particularly relevant as it pertains to refining tools and techniques for data acquisition and analysis. In the realm of neuroscience where understanding the intricacies of brain signals is paramount, our research topic offers a platform for exploring novel methodologies and exchanging ideas that could drive the field forward.

By delving into the complexities and nuances of biomedical data analysis, our research topic strives to foster collaboration, spark innovative approaches, and ultimately contribute to the collective advancement of knowledge in these critical scientific fields. 
